# The micro-architecture of human cancellous bone from fracture neck of femur patients in relation to the structural integrity and fracture toughness of the tissue

**DOI:** 10.1016/j.bonr.2015.10.001

**Published:** 2015-10-05

**Authors:** C. Greenwood, J.G. Clement, A.J. Dicken, J.P.O. Evans, I.D. Lyburn, R.M. Martin, K.D. Rogers, N. Stone, G. Adams, P. Zioupos

**Affiliations:** a Cranfield Forensic Institute, Cranfield University, Defence Academy of the UK, Shrivenham, UK; b Forensic Odontology, Melbourne Dental School, University of Melbourne, Melbourne, Australia; c The Imaging Science Group, Nottingham Trent University, Nottingham, UK; d Cobalt Health, Cheltenham, UK; e Social and Community Medicine, Bristol University, Bristol, UK; f Physics and Astronomy, Exeter University, Exeter, UK

**Keywords:** Osteoporosis, Microarchitecture, Fracture toughness (FT), Computed tomography (CT), Bone mineral density (BMD)

## Abstract

Osteoporosis is clinically assessed from bone mineral density measurements using dual energy X-ray absorption (DXA). However, these measurements do not always provide an accurate fracture prediction, arguably because DXA does not grapple with ‘bone quality’, which is a combined result of microarchitecture, texture, bone tissue properties, past loading history, material chemistry and bone physiology in reaction to disease. Studies addressing bone quality are comparatively few if one considers the potential importance of this factor. They suffer due to low number of human osteoporotic specimens, use of animal proxies and/or the lack of differentiation between confounding parameters such as gender and state of diseased bone. The present study considers bone samples donated from patients (n = 37) who suffered a femoral neck fracture and in this very well defined cohort we have produced in previous work fracture toughness measurements (FT) which quantify its ability to resist crack growth which reflects directly the structural integrity of the cancellous bone tissue. We investigated correlations between BV/TV and other microarchitectural parameters; we examined effects that may suggest differences in bone remodelling between males and females and compared the relationships with the FT properties. The data crucially has shown that TbTh, TbSp, SMI and TbN may provide a proxy or surrogate for BV/TV. Correlations between FT critical stress intensity values and microarchitecture parameters (BV/TV, BS/TV, TbN, BS/BV and SMI) for osteoporotic cancellous tissue were observed and are for the first time reported in this study. Overall, this study has not only highlighted that the fracture model based upon BMD could potentially be improved with inclusion of other microarchitecture parameters, but has also given us clear clues as to which of them are more influential in this role.

## Highlights

• first time ever study to relate microarchitecture to the fracture toughness of cancellous bone from the femoral head of FNF victims
• reduction in bone mass relates to a reduction in the number of trabeculae and trabecular thickness and an increase in trabeculae spacing
• bone loss observed appears to be a consequence of thinning of the trabeculae in males and perforation of the trabeculae in females
• study hints that TbTh, TbSp, SMI and TbN may provide a proxy or surrogate for BV/TV
• fracture models can be improved by including microarchitecture, BMD and the bone mineral quality of osteoporotic cancellous bone

## Nomenclature/glossary

CT: computed tomography.
_a_BMD: areal bone mineral density (as derived from DXA).
_v_BMD: volumetric bone mineral density (as derived from CT).
BS/BV: bone specific surface area (mm^− 1^).
BV/TV: bone volume/tissue volume.
DXA: dual energy X-ray absorptiometry.
FNF: fracture neck of femur.
FT: fracture toughness (MPa m^1/2^).
TbTh: trabecular thickness (mm).
TbSp: trabecular spacing (mm)
TbN: trabecular number (mm^− 1^).
TMD: tissue mineral density (g cm^− 3^) from CT values.
OP: Osteoporosis.
QUS: quantitative ultrasound.

## 1 Introduction

Osteoporosis is a multifactorial skeletal condition characterised primarily by a loss in bone mass (a bone quantity issue) and a constellation of other bone quality changes resulting in increased bone fragility. Osteoporosis is believed to affect around three million people in the UK alone (National Osteoporosis Society) and 200 million worldwide (International Osteoporosis Foundation).One in three women and 1 in 5 men over the age of 50 worldwide will suffer a fracture due to poor bone health (International Osteoporosis Foundation). Currently, the diagnosis and consequently the prevention and treatment of osteoporosis is assessed using dual energy X-ray absorption (DXA). DXA measures areal bone mineral density (_a_BMD) which confounds bone ‘quantity’ and mineral physical density. However, DXA does not provide unique information on ‘bone quality’, which is characterised by both the architecture of bone tissue and its chemistry. Cancellous bone strength is a corollary of its structural integrity which is a result of bone quantity, the quality of its bone matrix and its microarchitecture (Kleerekoper et al., 1985, Snyder et al., 1993).

Over the last decade, there is a gradual and increasing awareness that ‘bone quality’ may offer added understanding on the consequences of osteoporosis as a disease, for its diagnosis, prognosis and finally in its clinical management of the fractures in the hospital wards. Once the importance of bone quality has been highlighted various studies, on biopsies in-vitro, have shown that there are effects in the bone tissue material that accompany changes in bone mass density (Zioupos et al., 2008). Although measuring the same effects in-vivo is a major technological challenge, sincere efforts in this direction are being made and studies are becoming increasingly widespread. The advantage of obtaining data on the microarchitectural parameters of bone in addition of bone density alone (Müller et al., 1994, Laib et al., 1997) is evident by the fact that it claims to add to the power of a predictive relationship (Turner et al., 1990, Hildebrand et al., 1999, Nazarian et al., 2007) by increasing the R^2^ value from the usually reported levels of 0.50–0.60 (bone density alone) to 0.80–0.90 (after adding microarchitectural data).

Recent technological advances, most notably advances in high resolution in vivo computed tomography (CT) (Nishiyama and Shane, 2013, Liu et al., 2010a), may allow the in-vivo assessment of the microarchitectural properties of bone. Consequently, it should be expected that detailed characterisation of the bone microarchitecture can be combined to BMD at the level of a clinic and used to elucidate further the potential for fracture risk. This highlights the need for further studies to investigate the variance of bone tissue architecture, bone density and the macro-mechanical properties of bone, with the various parameters (BMD, strength, stiffness, toughness, microarchitecture, etc.) analysed either as separate characteristics and with the progress of the disease/condition, or by comparing them to each other. For applications in the clinical context this requires bespoke and well-defined cohorts and in studies where associated clinical data and scanning by other modalities is also available.

In-vitro laboratory studies of utilising CT to explore the effects of microarchitecture have been made on normal and ovariectomised animal proxies (Mittra et al., 2005, Teo et al., 2006, Kreipke et al., 2014) and these explored the relationship between microarchitecture and mechanical properties of cancellous bone as well as alterations to trabecular microarchitecture following ovariectomy. There only a few studies utilising human biopsies and these suffer from low sample numbers possibly due to the difficulty of obtaining substantial amount of human osteoporotic bone material (Zhang et al., 2010, Green et al., 2011). Studies that examine the mechanical and architectural properties of human bone often do not screen for bone diseases such as osteoporosis and osteoarthritis and/or do not differentiate between ‘normal’ and osteoporotic bone (Ulrich et al., 1999, Perilli et al., 2008, Wu et al., 2013, Thomsen et al., 2015) and/or they do not consider the differences between males and females (Wu et al., 2013, Link et al., 1998, Ding and Hvid, 2000, Follet et al., 2011). This highlights a clear need to study the tissue of an osteoporotic cohort with sufficient numbers of samples to provide statistically meaningful results and valuable insights into the cause/effect relationship and the mechanobiology of bone. In the present study we had access to a significant collection of samples from fracture neck of femur (FNF) patients from a large study on cancellous bone integrity by invasive and non-invasive means (Cook and Zioupos, 2009, Cook et al., 2010). These samples have been mechanically characterised at the macroscopic level (macromechanical in-vitro tests (Cook and Zioupos, 2009) and have also been associated with clinical measurements of bone status in-vivo by quantitative ultrasound (QUS) tests applied on the calcaneus of these same patients (Cook et al., 2010). The mechanical analysis, in particular, applied to these samples is unique in that it contained for the first time ever a characterisation of the fracture toughness of the samples considered to be a better measure of the resistance of bone to fracture than other conventional tests. Consequently quantification of the microarchitecture of human cancellous bone by means of the currently well-established histomorphometric parameters such as porosity (BV/TV), trabecular number, thickness and spacing, degree of anisotropy offers two distinct benefits: (1) a definite range for their values in a well-defined population and (2) correlation to novel mechanical characterisation measurements at the macroscale. It is self-evident that the higher the quality of the population cohort is (definition, selection, screening, medical history, etc.), the higher is the standard of the microarchitecture values and the more enabling such parameters will be in elucidating the underlying factors of the disease. Furthermore, linking them to meaningful mechanical properties may provide an insight into whether any of these architectural signatures can in themselves be linked to potential fracture risk.

## 2 Materials and methods

### 2.1 Bone specimens

A sample set of 37 femoral heads were collected from osteoporotic patients who had suffered trauma fractures at the femoral neck and consequently required hip replacement surgery. Population characteristics are provided in Table 1. Ethical approval for the collection and use of these specimens was provided by Gloucestershire NHS trust REC (acknowledgments).

### 2.2 Specimen preparation

Specimen preparation (including sectioning from the femoral head and cleaning) has previously described in detail (Cook and Zioupos, 2009, Cook et al., 2010). Single edge notched beam samples (SENB) were prepared to quantify fracture toughness by assessing the necessary stress conditions to start the advancement of a crack from a man-made notch. Because the notch can grow a crack in between (along) trabeculae, or across trabeculae, we attempted to cut SENB samples in at least one (along), and where possible both, directions (along & across) the trabecular network in each sample. All specimens were kept frozen at − 20 °C prior to sample preparation. The sectioning was performed by using a metallurgical saw (Struers® Accutom-2), they were then sanded and polished by using progressively finer grades of carbide paper (400–2500 grit) to the dimensions required for material testing. Specimens were manufactured in the shape of beams (30 × 6 × 3 mm) for mechanical material testing as single edge notch beam samples (SENB). Sample preparation was performed under constant water irrigation, to prevent the production of microcracks or other damage to the specimens.

### 2.3 Micro-computed tomography

The specimen microarchitecture was examined with micro computed tomography (μCT). Each specimen was scanned using a Nikon CT H225 (X-Tek Systems Ltd., Tring, Hertfordshire, UK) cone beam μCT scanner operated at. 35 kV, and 115 μA. The geometric magnification produced, a voxel dimension of ~ 15 μm. Noise reduction and beam hardening corrections were applied to the data and VG Studio Max 2.2 (Volume Graphics GmbH, Heidelberg, Germany) utilised to visualise and quantify several microarchitectural features. These included trabecular thickness (TbTh), spacing (TbSp) and number (TbN), surface area (BS), material volume (BV) and total volume (TV). QRM MicroCT-HA (QRM GmbH, 91,096 Möhrendorf, Germany) calibration phantoms, which differed in known tissue mineral density values (_v_TMD), were scanned and reconstructed under the same conditions as the specimens. The mean grey scale values taken from the attenuation histograms for these phantoms were then used to construct a calibration curve of _v_TMD values and grey scales. This allowed calculation of tissue mineral density values for the trabecular specimens. _v_TMD values were then used to determine bone mineral density values (_v_BMD) according to:

(1) $${}_{v}\mathrm{BMD}={\;}_{v}\mathrm{TMD}\times \mathrm{BV}/\mathrm{TV}.$$

In this article we have elected to use the subscript ‘v’ next to _v_BMD and _v_TMD for the case where these measures are calculated on volumetric basis in a microCT machine. Bone mineral density values as derived by DXA are conventionally measured over unit of area and for these, to make the distinction, we will use the subscript ‘a’ as in _a_BMD. In the case of determining tissue mineral density there are of course, other alternative methods such as those using Archimedes principle to calculate volume and ashing or dissolving to calculate mineral mass, in which case it is also important to denote our present values as _v_TMD for clarity. BoneJ© [http://bonej.org/; http://rsbweb.nih.gov/ij/] was employed at a second stage to calculate additional micro-architectural parameters such as structure model index (SMI) and degree of anisotropy (DA).

### 2.4 Mechanical testing

**Fig. 1:**
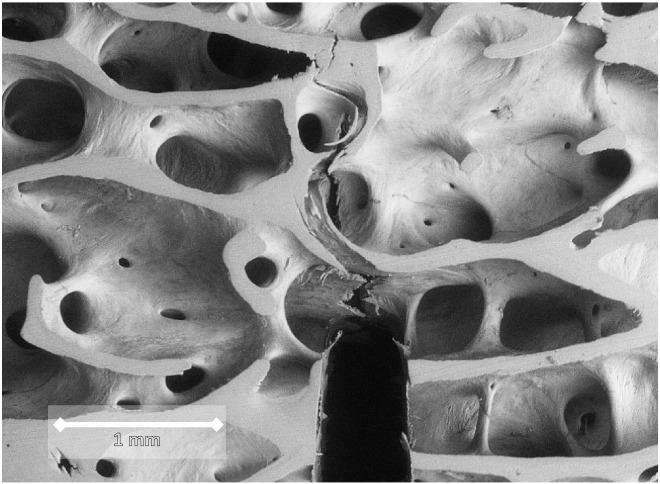
Crack growth emanating from the man-made notch as in the SENB FT tests of Cook and Zioupos (2009) and Ding and Hvid (2000). Following application of bending moments on either side the notch opens up and the trabeculae ahead of it start snapping. The deformation and crack growth was stopped as soon as this first critical load was reached. The load was used to define the K_c_ value (initial notch width ~ 300 μm.).

The SENB (beam shaped) samples have been mechanically characterised in previous papers for fracture toughness in linear elastic FM approach. The K_c_ values were derived for the load at a point where the man-made notch started growing (following extensive yielding and bending of the trabeculae ahead of the notch) by snapping of one or more trabeculae in the first instance (Fig. 1). The deformation was measured by a miniature extensometer (Model 3442-006M-050ST) attached at the mouth of the notch. The dimensions and other restrictions that were followed complied with the usual material testing standards such as ASTM E399-90 as reported in Cook & Zioupos 2009 (Cook and Zioupos, 2009). The mechanical testing was undertaken using a Dartec Series HC25 materials testing machine (Zwick ltd., Leominster HR6 0QH, UK) driven by a 9610 series controller unit and operated using Workshop 96© software. Load was monitored using a 500 N load cell (RDP Electronics Ltd., Wolverhampton WV10 0PY, UK) whilst the gauge length of the crack mouth opening displacement measured by the extensometer was 6 mm. The loading rate during fracture toughness testing was 0.05 mm s^− 1^ (3 mm min^− 1^), with Data Acquisition at a capture rate of 1000 points per minute. Unlike studies of the past which tested cancellous bone by applying compression on bone cylindrical cores or cubic shaped samples, these test were the first ones ever to attempt a quantification of the necessary loading conditions that would allow a crack to start to grow from stability into an unstable fracture mode. In this respect the mechanical data offers a novel and invaluable way of assessing the structural integrity and loading ability of these samples in a way that resembles the conditions in FNF situations in a more biofidelic manner.

### 2.5 Statistical analysis

Statistical analysis was carried out to determine the differences in the microarchitecture and density parameters between males and females. Anderson–Darling tests were carried out to determine whether the data values are normally distributed. In cases of normal distribution, a 2 sample t-test was carried out to determine statistical significance between males and females. The non-parametric equivalent of the t-test, the Mann–Witney test was performed on non-normally distributed data. Pearson's correlation coefficients were used throughout the study. R^2^ values reported are those that have already been adjusted for the degrees of freedom (i.e. taking into account the number of samples per test). For each parameter the intra-subject and inter-subject variability was examined and depending on the nature of the parameter and the observed variance the data was either averaged for each donor or held up on each own on a sample per sample basis. Mechanical parameters, for instance, had to be examined on a sample per sample basis because anisotropy due to the direction has a more profound effect than differences between donors caused by the porosity level.

## 3 Results

### 3.1 Micro-architecture

As shown in Table 2, a wide range of parameters were considered to investigate the micro-architecture of osteoporotic specimens obtained from both females and males. The P-values from the statistical analysis of the mineral architecture parameters between males and females are also provided in Table 2, as well as values reported within previous studies. Overall values were not significantly different between males and females when comparing the specimens from this study for BV/TV, DA, SMI and _v_BMD. In contrast, BS/BV, TbTh, TbSp, TbN, and TMD values were significantly different between the two groups. When compared to other literature, the average values for BV/TV, BS/BV, _v_BMD, TbSp and TbN presented in this study are within the same range as those previously reported; while for TbTh values presented in this paper are slightly lower, and DA, SMI and TMD are higher than the literature (Green et al., 2011, Ulrich et al., 1999, Perilli et al., 2008, Wu et al., 2013, Thomsen et al., 2015, Link et al., 1998, Ding and Hvid, 2000, Macdonald et al., 2011, Liu et al., 2010b, Milovanovic et al., 2012, Meunier and Boivin, 1997, Humbert et al., 2012). However, our population are exclusively osteoporotic and the aforementioned values have shown a trend one way or another with the progress of the disease. Study of age dependence showed that age was not a significant confounding factor in any of the subsequent analysis.

**Fig. 2:**
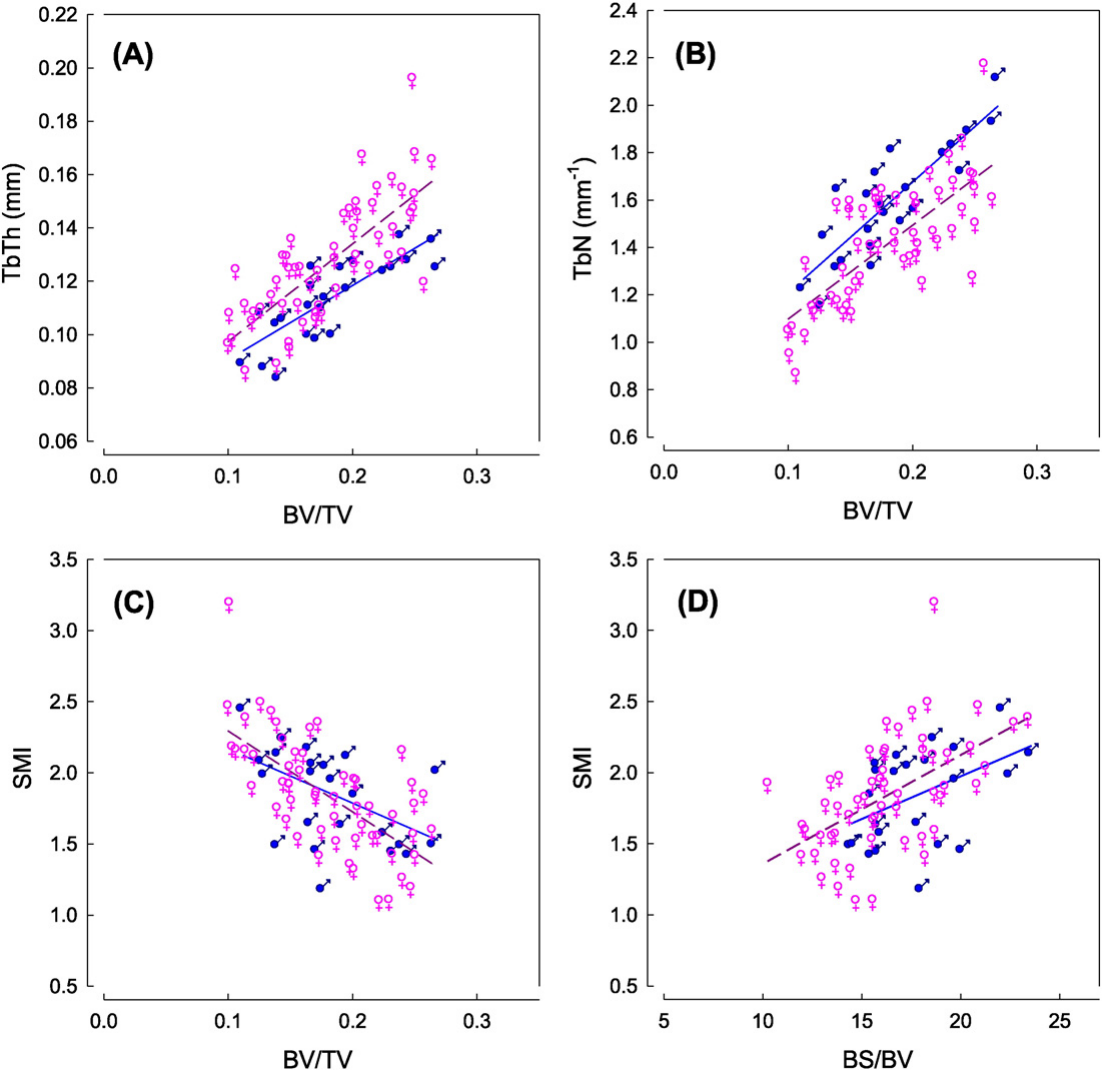
Behaviour in-between the various microarchitectural parameters: TbN, TbTh, SMI vs BV/TV and BS/BV. Samples are tagged for male (♂) and female(♀). Least squares regression lines are shown for male (solid) and female (dashed).

The relationship between the quantity of bone, BV/TV, (which is used in bone mineral density calculations) and other mineral architecture parameters was investigated for this study to provide an overview of the nature of osteoporosis as measured by micro-CT. As shown in Fig. 2A, a strong positive correlation between TbTh and BV/TV was observed. A similar relationship was also observed for TbN (Fig. 2B), indicating that with increasing TbTh, TbN the quantity of bone material increases. The quantity of bone mineral is also correlated to SMI values (Fig. 2C). Higher SMI values, which may indicate that trabeculae are more rod-like in structure, corresponds to a lower quantity of bone material. SMI values also correlated to specific surface area values (BS/BV) as shown in Fig. 1D, with more rod-like trabecular structures correlating to larger specific surface areas. No correlation was observed between bone quantity (BV/TV) and DA. Differences between the TbTh and TbN values for males and females are highlighted in Figs. 2A and 2B. On average, the TbTh for males is lower than females, whilst TbN is greater in males than females. No significant difference in SMI is observed between males and females. Correlation coefficients and levels of statistical significance observed when BV/TV is a function of each microarchitecture parameter are reported in Table 3. Correlation coefficients and levels of statistical significance observed in particular when BS/BV is a function of SMI are also reported.

### 3.2 Micro-architecture and mechanical properties

**Fig. 3:**
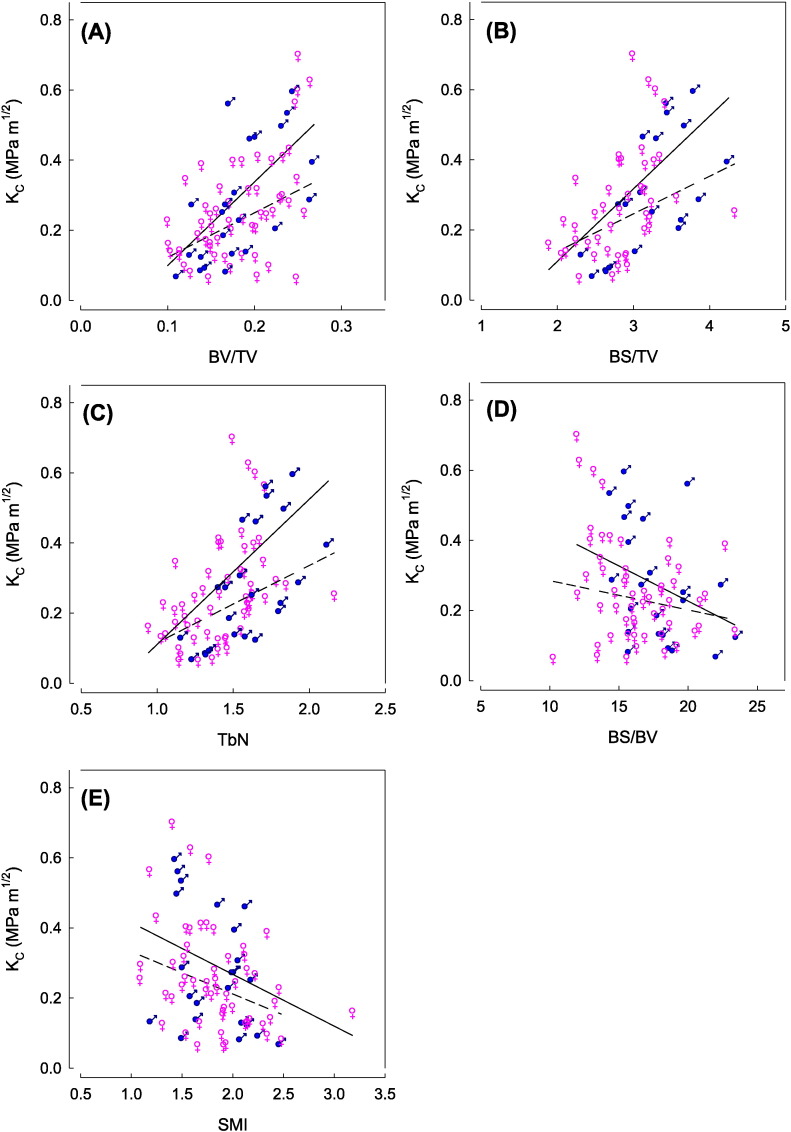
Critical stress intensity (K_C_) as a function of various microarchitectural properties: (A) BV/TV; (B) BS/TV; (C) TbN; (D) BS/BV; (E). SMI. Samples are tagged for male (♂) and female(♀). Toughness values depend on the orientation of the man-made crack with respect to the trabeculae. In each plot two regression lines are shown for the crack running across the trabeculae (stronger/tougher behaviour: solid line -) and along/in-between the trabeculae (softer/weaker behaviour: dashed line —). In all regressions the slope constants were significantly different from zero (P < 0.05) showing a statistically significant effect of all 5 parameters vs K_C_. However, because of the noise/scatter in the data the behaviour with respect to the two orthogonal directions (across vs along the trabeculae) was different only for (A) K_C_ vs BV/TV and (C) K_C_ vs TbN. The errors in each data point have been excluded from the graphs for clarity. The mean errors are as follows: BV/TV: 0.06, BS/TV: 0.21, TbN: 0.06, BS/BV: 0.50 and SMI 0.29.

The architectural properties of osteoporotic bone and its corresponding mechanical properties calculated from fracture toughness testing are herein reported for the first time. The critical stress intensity values (K_C)_ correlated to a number of the micro-architectural properties of the specimens. A positive correlation was observed between K_C_ and BV/TV (Fig. 3A), BS/TV (Fig. 3B), TbN (Fig. 3C) and TbSp. This suggests that with increasing bone quantity, surface area, trabecular number and trabecular spacing, the amount of force required to fracture the specimen increases. A negative correlation between K_C_ and BS/BV (Fig. 3D) suggests as the specific surface area increases, the critical stress intensity decreases. This relationship was also observed for SMI (Fig. 3E), suggesting as the trabeculae become more rod-like in structure, less force is required to fracture the specimen. No correlation was observed between K_C_ and TbTh or DA. Pearson's correlation coefficients and the P values observed between the critical stress intensity (K_C_) and each microarchitecture parameter are reported in Table 4.

### 3.3 Material density and mechanical properties

**Fig. 4:**
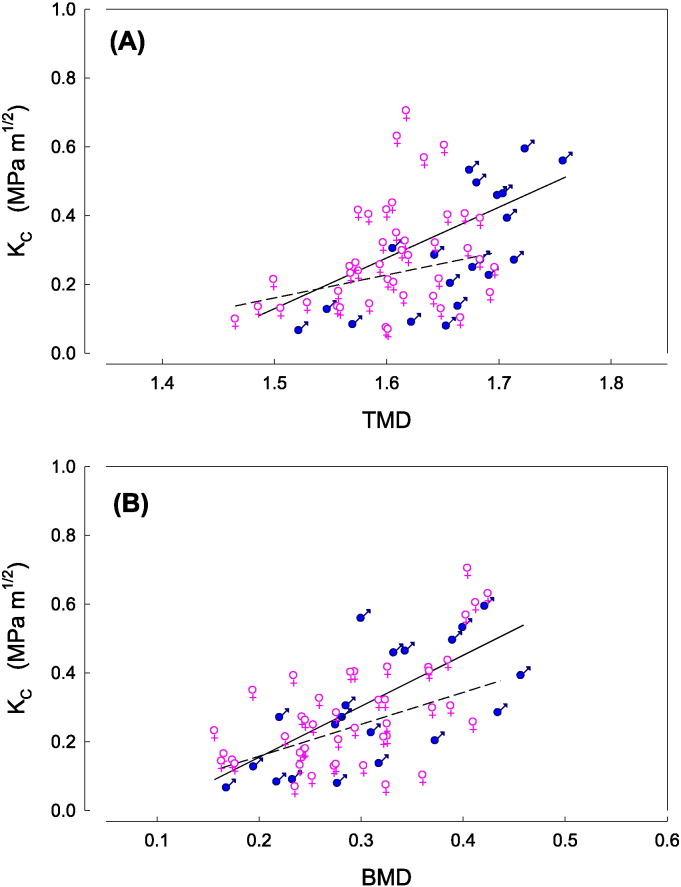
Critical stress intensity (K_C_) as a function of (A) TMD; and (B) _v_BMD. Regression lines are shown for the crack running across the trabeculae (stronger/tougher behaviour: solid line -) and along/in-between the trabeculae (softer/weaker behaviour: dashed line —). Regressions in: the direction across trabeculae: K_C_ = − 2.08^⁎⁎^ + 1.48^⁎⁎^ TMD (R^2^_adj_ = 0.32); K_C_ = − 0.142^†^ + 1.48** _v_BMD (R^2^_adj_ = 0.49). In the direction along trabeculae: K_C_ = − 0.844 + 0.669 TMD (R^2^_adj_ = 0.03); K_C_ = − 0.028 + 0.927* _v_BMD (R^2^_adj_ = 0.20). Levels of significance: **P < 0.01; *P < 0.05; ^†^P < 0.1.

As an adjunct to the architectural and mechanical studies, we also considered bone material quality characteristics through attenuation in material density. A positive correlation was observed between the tissue mineral density (_v_TMD), and K_C_ for both males and females (Fig. 4A). On average, larger _v_TMD values were observed for males than females. The same correlation (Fig. 4B) was observed for bone mineral density (_v_BMD), which not only encompasses _v_TMD but also the quantity of bone material (BV/TV). Fig. 4B also highlights that there is no distinction between males and females when considering _v_BMD values.

## 4 Discussion

The present article outlines a detailed study of the micro-architecture, the bone quality and the associated structural integrity of cancellous bone specimens collected from a cohort of patients who had suffered fracture of the neck of femur and therefore had been admitted for emergency surgery for hip replacement. The collection of samples is unique in the sense that the mechanical results, presented in other articles in the past (Cook and Zioupos, 2009, Cook et al., 2010), were the only ones in the literature that reported fracture toughness properties of the bone and in both its two predominant (practically orthogonal) directions that reflected fracture across and along the main trabecular scaffold (Cook and Zioupos, 2009). Modern CT scanners allow a detailed numerical analysis of the geometry and architecture of the trabecular network and add a wealth of information to the usual DXA scans, which practically only grapple with cancellous density. Density is the most influential factor as far as the structural integrity and mechanical competence of cancellous bone is concerned (R^2^ values of mechanical performance versus density are in the order of the 0.50 to 0.70 in the best of cases). However, it is now universally believed that adding other histomorphometric values which express the full architecture of the tissue (those that currently DXA does not capture) would greatly increase the predictive power of any such relationships (Snyder et al., 1993, Turner et al., 1990, Ulrich et al., 1999), which are based simply on density to start with.

### 4.1 Cancellous architecture

The findings of the present study support a model of reduced bone mass going hand in hand with a reduction in the number of trabeculae and an increase in trabecular spacing as OP progresses within this cohort of patients. The present reported values are within the ranges reported within previous studies [Table 2]. Furthermore, consistent with previous studies of ageing bone the SMI values suggest that the trabeculae turn more rod-like in structure than plate-like with the progress of OP. This is thought to be a natural consequence of progressive loss of bone mass and has previously been reported as a significant age-related change (Ding and Hvid, 2000). The present SMI values are significantly greater than those previously reported in one study for femoral head specimens (Wu et al., 2013). They are however, lower than those values reported for specimens taken from the supralateral neck (Milovanovic et al., 2012) and trochanter (Wu et al., 2013). On the whole more than 70% of the present data has values greater than the max SMI previously reported. This may be either: (a) a consequence of the anatomic site (for instance, comparing samples from the neck vs head of femur), or (b) if true, it may reflect that bone remodelling in osteoporotic patients could differ to the remodelling observed in the normal ageing population. If the latter is true then SMI could potentially offer a valuable measure in the diagnosis of the severity of OP and/or when evaluating fracture risk. On geometric considerations alone, the high SMI values are in some respect consistent with high BS/BV values simply because one would expect a larger specific surface area for rod-like structures in comparison to plate-like structures.

The degree of trabeculae orientation (DA) is greater than that reported previously for studies that focused on age changes alone [Table 2]. This is consistent with the increased amount of bone loss in osteoporosis. Previous studies have suggested this bone loss occurs preferentially in non-mechanical loading areas (Ruff et al., 2006, Boyle and Kim, 2011) demonstrating a loss of the horizontal trabeculae in the femoral head (Fazzalari, 1993, Ding and Hvid, 2000) and consequently resulting in a greater degree of anisotropy. TbTh values for this study were somewhat smaller than those reported in previous studies. Once again if this is a true effect (not caused by methodology) it may reflect the fact that our cohort of donors had a higher than usual mean age (81.4 yrs) (Ding and Hvid, 2000, Hordon et al., 2000) and the fact that our donors were OP and had already sustained a fracture.

The change in microarchitectural properties as the bone volume changes may be used to refine the understanding of remodelling processes in osteoporotic bone. For example, differences in the trabecular thickness and number with relative bone volume (BV/TV in Fig. 2A and B, ANCOVA/P = 0.05), suggests a difference in the remodelling process between male and female donors. For the same amount of BV/TV the data (observe the relative position of the regression lines above and below each other) supports the view (Aaron et al., 1987, van der Linden et al., 2001, Seeman, 2008) that mineral volume in males reduces through trabeculae thinning, whilst for females perforation of the trabeculae is the dominant mechanism. Furthermore, as bone mass decreases, trabeculae become more rod-like and the specific surface area increases. It is reasonable to propose that there is an acceleration of bone loss that is inversely dependent on the volume of mineral.

The correlations between BV/TV vs. TbTh, TbSp, TbN and SMI were all significant (P < 0.01), and although these parameters interdepend on each other (linked by the progress of the disease) it is perfectly feasible that this set of microarchitectural parameters may provide a proxy or surrogate for BV/TV. This may be a valuable option as the calculation of BV/TV is difficult in-vivo, and even in-vitro it is time and resource intensive as demonstrated when using imaging techniques such as dissection, microscopy, or Archimedes principle. Provided modern CT scanner can produce the resolution to allow quantification of TbTh, TbSp, TbN and SMI in-vivo, a proxy for the quantity of bone mass would potentially prove very beneficial.

### 4.2 Micro-architecture and mechanical properties

The current specimens has been already characterised for fracture toughness for cracks growing in the two predominant directions across and along the trabecular network. The R^2^ values for correlation of K_C_ vs BV/TV were in the order of values reported previously on other mechanical studies for cancellous strength and stiffness vs BV/TV (Perilli et al., 2008). In addition to the previous studies, the present BS/TV values show that there is also a greater mechanical strength the greater bone surface area there is. However, the same is not true for specific surface area (BS/BV), whose values are arguably also influenced by the form, shape and size of the trabeculae. The negative linear correlation between K_C_ and SMI suggests that as the trabeculae tend towards a more rod-like structure, the critical stress intensity decreases. Notwithstanding the significant directional effects and implications of combining plates interlaced with rods, as previous research has shown, trabecular plates are stiffer and more resistant to fracture in at least 2 directions (i.e. along their plane) and easily subject to buckling across their plane when the supporting rods collapse. Meanwhile a trabecular network consisting of rods is less constrained and more susceptible to yielding in 3D (Garrison et al., 2009). Thus overall greater energy absorption may be observed for plate-like trabeculae in comparison to rod-like trabeculae. Consequently, Bevill et al. (2006) reported that one of the primary factors associated with the failure of trabeculae bone is the transition from plate-like to rod-like structures. However, the correlation between SMI and K_C_ is not particularly strong (R^2^ = 0.14) indicating that it cannot be considered a primary qualitative cause for toughness. Although SMI provides a qualitative account of shape of the trabeculae (plates vs rods), it does not accommodate the actual number of trabeculae. The same limitation may also account for the poor correlation between TbTh and K_C_. For example, specimens with large numbers of thin trabeculae and those with smaller numbers of thicker trabeculae may possess similar K_C_ values and therefore cannot be differentiated on TbTh alone. Given the noise caused by biological variability samples with similar BV/TV can vary noticeably in TbTh.

Not surprisingly perhaps, there was no measurable correlation between the degree of trabeculae orientation and K_C_. Because the samples were anisotropic K_C_ was measured in one of 2 directions in each sample. A relationship to correlate DA and mechanics would have to be produced from a single sample measured in 2 directions and related to the associated DA value for that sample. This is simply not possible. However, it is worth commenting that there is a theory to account for inter-effects between DA and mechanics and the degree of OP. It has previously been reported that preferential dissolution and loss of ‘horizontal’ trabeculae occurs in vertebral bone during ageing (Ding and Hvid, 2000), whilst the vertical trabeculae in vertebral bone have been shown to increase in thickness with age (Thomsen et al., 2015). If this preferential thickening and dissolution occurs within the femoral head, the degree of anisotropy will increase possibly decreasing the amount of energy required to drive a crack in the direction in-between the trabeculae and this will increase the perceived mechanical anisotropy. The R^2^ values presented herein for K_C_ and the architectural properties are generally slightly lower than some of those that have been reported for stiffness and strength. We cannot read too much into this as invariably our values for both mechanical and architectural parameters are simple averages over the entire specimen. A number of studies have reported stronger correlations between mechanical properties or physicochemical properties and architectural parameters when properties are considered locally in sites within the trabeculae network. This is to be expected. In our study it is reasonable to expect that if the precise architectural and bone material condition ahead of the notch were examined even better correlations would be achieved. However, this would detract from its use as a study for in vivo analysis and assessment of osteoporosis and fracture prediction. Overall we are confident that the control we exerted on the factor ‘direction’ in our tests, plus the fact that true fracture toughness properties are presented, allows us to have a clearer view of the course and the consequences of the osteoporotic condition of these patients relatively to many other studies on this topic.

### 4.3 Density and mechanical testing

A significant novel element of our present study is the data we present on tissue mineral density (_v_TMD) alongside bone mineral density (_v_BMD). An increase in _v_TMD during ageing has been previously reported in the literature either directly (Green et al., 2011, Follet et al., 2011, Meunier and Boivin, 1997) or indirectly (Zioupos et al., 2008). The values reported here are significantly lower than in previous human tissue studies (Green et al., 2011, Follet et al., 2011), but we attribute this to the evident osteoporotic condition of the bone specimens utilised in this study. Previous literature has demonstrated a decrease in TMD in ovariectomised baboons and suggested that this was due to an increase in remodelling activity (Meunier and Boivin, 1997). In such cases one must always clarify whether this refers to the relative fraction of mineral mass to the organic mass. An decrease in TMD as observed here must be caused either by a decrease in bone mineral mass, per se, in each unit of volume scanned (voxel); or by a different bone chemistry for females and males. On average, the TMD for males was significantly greater than females, which may suggest three possibilities: (i) initial TMD for males is significantly greater for males than females, (ii) a different ratio of mineral to organic loss in males than females, or (iii) there is difference in the collagen and/or mineral chemistry (Boskey et al., 2005, Faibish et al., 2006, Gamsjaeger et al., 2014); or a combination of all three factors. Further work would be required to elucidate these possibilities.

As expected a positive correlation between _v_BMD and K_C_ was observed and in fact a much better correlation between _v_BMD and K_C_ in comparison to _v_TMD. This is a simply a result of the fact that _v_BMD combines both bone quantity in BV/TV and bone quality in _v_TMD as opposed to bone quality alone (_v_TMD). For fracture risk prediction it is evident that it is necessary to take into consideration both the quantity of bone material (BV/TV) and TMD. The DXA machines currently in use do produce an equivalent measure in _a_BMD, which is mineral density value over a surface area (not volumetric) and results in the physics sense from X-ray absorption as a result of both bone quantity (mineralised matrix per pixel) and bone quality (brightness per pixel). However, as shown in Fig. 4B, even the present volumetric _v_BMD, which was produced in-vitro with a high resolution and at laboratory standards conditions, does not perfectly correlate to K_C_. This may explain why presently _a_BMD calculations often fail to predict impending fractures. As the promising correlation coefficients (R^2^) show for relationship between _v_BMD vs K_C_ one valid rationale for future exploratory studies would be to improve the accuracy of bone mineral density as a clinical predictor of fracture and perhaps add architectural parameters to strengthen further the predictive power.

## 5 Conclusion

This study has for the first time considered the relationship between microarchitecture and mechanical properties for a considerable number of cancellous bone specimens collected from a cohort of patients who had suffered fracture of the neck of femur. The findings support a model where reduced bone mass results in a reduction in the number of trabeculae and trabeculae thickness and an increase in trabeculae spacing. This is further combined to a transformation from plate like to rod like trabeculae structure, validated by both SMI and BS/BV values. Remodelling differences between males and females have been highlighted and validate the current view that bone loss observed in males is mainly a consequence of thinning of the trabeculae whilst in females it is the perforation of the trabeculae. This study has also crucially shown that TbTh, TbSp, SMI and TbN may provide a proxy or surrogate for BV/TV. Correlations between critical stress intensity factor values and microarchitecture parameters (BV/TV, BS/TV, TbN, BS/BV and SMI) for osteoporotic cancellous tissue are for the first time reported in this study. Further work is required to identify why the TMD values reported for our osteoporotic population are lower than previously reported, including an investigation of the related collagen and mineral chemistry. Overall, this study has not only highlighted that the fracture model based upon BMD could potentially be improved with inclusion of other microarchitecture parameters, but has also highlighted the hidden role of the underlying microarchitecture parameters and bone mineral quality in osteoporotic cancellous bone.

## Disclosures

There is no conflict of interest to declare.

## Figures and Tables

**Table 1:** Demographic characteristics of the cohort of FNF donors. Two thirds of the patients were in the 80–91 years range.

Donors	37
Male/Female	7/30
Number of specimens	23/57
Age range (years)	59–96
Age mean (years)	82.3 (SD = 6.8)
Weight range (kg)	41.3–82.6
Weight mean (kg)	64.2 (SD = 10.5)
Height range (m)	1.55–1.80
Height mean (m)	1.67 (SD = 0.08)

**Table 2:** Average values of the microarchitectural properties for samples in males and females. Standard deviation values are also provided. P-values denoting significant differences (P < 0.05) between males and females are included. Microarchitecture values from other studies are also provided for comparison.

Parameter	Females (n = 57)		Males (n = 23)		P-values	Range in the literature	References
	Mean	Std. dev.	Mean	Std. dev.			
BV/TV	0.18	0.05	0.18	0.04	0.92	0.16–0.30	Green et al., 2011, Perilli et al., 2008, Wu et al., 2013, Thomsen et al., 2015, Link et al., 1998, Macdonald et al., 2011
BS/BV (mm^− 1^)	16.12	3.00	17.84	2.49	0.01	10.54–17.8	Zhang et al., 2010, Liu et al., 2010a, Liu et al., 2010b
TbTh (mm)	0.13	0.03	0.11	0.02	0.01	0.16–0.25	Green et al., 2011, Perilli et al., 2008, Wu et al., 2013, Thomsen et al., 2015, Ding and Hvid, 2000
TbSp (mm)	0.60	0.14	0.52	0.10	0.03	0.36–0.82	Green et al., 2011, Perilli et al., 2008, Wu et al., 2013
TbN (mm^− 1^)	1.42	0.25	1.60	0.24	< 0.01	1.45–2.11	Green et al., 2011, Perilli et al., 2008, Ding and Hvid, 2000
DA	3.27	1.07	3.34	1.08	0.61	1.73–2.00	Green et al., 2011, Wu et al., 2013
SMI	1.81	0.44	1.85	0.33	0.72	0.50–2.61	Green et al., 2011, Wu et al., 2013, Ding and Hvid, 2000, Milovanovic et al., 2012
TMD (g HAcm^− 3^)	0.77	0.04	0.80	0.05	< 0.01	0.72–1.25	Green et al., 2011, Follet et al., 2011, Meunier and Boivin, 1997
_v_BMD (g/cm^− 3^)	0.29	0.08	0.31	0.08	0.40	0.22–0.37	Macdonald et al., 2011, Humbert et al., 2012

**Table 3:** Pearson's correlation coefficients and P-values for correlations observed when BV/TV is a function of each microarchitecture parameter and for the case of BS/BV vs SMI.

	Females		Males	
	R^2^ values	P-values	R^2^ values	P-values
BS/BV	0.63	< 0.01	0.61	< 0.01
TbTh	0.58	< 0.01	0.67	< 0.01
TbN	0.55	< 0.01	0.74	< 0.01
TbSp	0.64	< 0.01	0.80	< 0.01
SMI	0.42	< 0.01	0.26	< 0.01
DA	0.07	0.53	0.08	0.20
BS/BV vs SMI	0.31	< 0.01	0.21	0.03

**Table 4:** Pearson's correlation coefficients and P-values for correlations observed when critical stress intensity (K_C_) is a function of each microarchitecture parameter.

	Females		Males	
	R^2^ values	P-values	R^2^ values	P-values
BV/TV	0.29	< 0.01	0.41	< 0.01
BS/BV	0.12	0.01	0.17	0.05
TbTh	0.20	< 0.01	0.19	0.04
TbN	0.23	< 0.01	0.4	< 0.01
TbSp	0.25	< 0.01	0.44	< 0.01
SMI	0.14	< 0.01	0.13	0.09
DA	0.07	0.05	0.20	0.04
_v_TMD	0.08	0.07	0.52	< 0.01
_v_BMD	0.36	< 0.01	0.45	< 0.01
